# *Methods and Protocols*—Aims and Scope Update

**DOI:** 10.3390/mps6060111

**Published:** 2023-11-17

**Authors:** Fernando Albericio, Philip Hublitz

**Affiliations:** 1 School of Chemistry and Physics, University of KwaZulu-Natal, Durban 4001, South Africa; 2 Evotec International GmbH, Marie-Curie-Straße 7, D-37079 Göttingen, Germany

As our readers know, *Methods and Protocols* is a multidisciplinary peer-reviewed scientific journal that provides a forum to the publication of novel approaches in the fields of Life Sciences, Chemistry, and Biomedical Sciences and their intersection with other related scientific fields such as Physics, Earth Sciences, and Environmental Research. Our principal aim is to provide scientists with a platform to publish new protocols based on established techniques as well as significant improvements and developments of cutting-edge methods. In line with our scientific scope, all articles need to provide background information on underlying principles, full experimental details, and comparison to available protocols.

We just celebrated our first successful official impact factor of 2.4 and we are proud to have established *MPs* as a respected journal. Along with this, we are lucky to attract an ever-growing stream of high-quality manuscripts. In our latest editorial team meeting, we decided to implement some changes to the kind of manuscripts *MPs* will consider for publication, and we have updated our editorial section. The highlights of these changes are summarized in the following. We encourage authors to submit full articles as classical research manuscripts, presenting novel information with all required controls and concepts. We encourage submissions of relevant review contributions in the classical sense as well as short comments highlighting active areas of research. In terms of protocols, we are eager to attract classical submissions that describe novel or sufficiently re-defined detailed step-by step descriptions of methodology. The impact of this is broadened by the new category “Technical Reports”, which can be significantly shorter but need to highlight technical novelty and applicability in the designated research area. A new structure is taken on board for contributions dealing with study design and prospective research proposals. We want to continue giving authors the opportunity to present their concepts in our newly established Registered Reports or Study Protocols Section (full details can be found on the new *MPs* website). The overarching principle should be that manuscripts must present data and data analysis in support of any new concept. A pure study protocol for feasibility studies or scoping reviews will no longer be in the focus of *MPs*.

We hope that these changes are well accepted among our authors and contributors. We hold the firm belief this scientific change of direction will increase the high level of quality of our accepted manuscripts and will help *MPs* to further grow into the right direction. We are very much looking forward to receiving many more wonderful contributions as methods, protocols, reviews, comments, technical notes, and data-supported study reports.

